# Global Distribution of Outbreaks of Water-Associated Infectious Diseases

**DOI:** 10.1371/journal.pntd.0001483

**Published:** 2012-02-14

**Authors:** Kun Yang, Jeffrey LeJeune, Doug Alsdorf, Bo Lu, C. K. Shum, Song Liang

**Affiliations:** 1 College of Public Health, The Ohio State University, Columbus, Ohio, United States of America; 2 Department of Schistosomiasis, Jiangsu Institute of Parasitic Diseases, Wuxi, People's Republic of China; 3 Ohio Agricultural Research and Development Center, Wooster, Ohio, United States of America; 4 School of Earth Sciences, The Ohio State University, Columbus, Ohio, United States of America; London School of Hygiene & Tropical Medicine, United Kingdom

## Abstract

**Background:**

Water plays an important role in the transmission of many infectious diseases, which pose a great burden on global public health. However, the global distribution of these water-associated infectious diseases and underlying factors remain largely unexplored.

**Methods and Findings:**

Based on the Global Infectious Disease and Epidemiology Network (GIDEON), a global database including water-associated pathogens and diseases was developed. In this study, reported outbreak events associated with corresponding water-associated infectious diseases from 1991 to 2008 were extracted from the database. The location of each reported outbreak event was identified and geocoded into a GIS database. Also collected in the GIS database included geo-referenced socio-environmental information including population density (2000), annual accumulated temperature, surface water area, and average annual precipitation. Poisson models with Bayesian inference were developed to explore the association between these socio-environmental factors and distribution of the reported outbreak events. Based on model predictions a global relative risk map was generated. A total of 1,428 reported outbreak events were retrieved from the database. The analysis suggested that outbreaks of water-associated diseases are significantly correlated with socio-environmental factors. Population density is a significant risk factor for all categories of reported outbreaks of water-associated diseases; water-related diseases (e.g., vector-borne diseases) are associated with accumulated temperature; water-washed diseases (e.g., conjunctivitis) are inversely related to surface water area; both water-borne and water-related diseases are inversely related to average annual rainfall. Based on the model predictions, “hotspots” of risks for all categories of water-associated diseases were explored.

**Conclusions:**

At the global scale, water-associated infectious diseases are significantly correlated with socio-environmental factors, impacting all regions which are affected disproportionately by different categories of water-associated infectious diseases.

## Author Summary

Water is essential for maintaining life on Earth but can also serve as a media for many pathogenic organisms, causing a high disease burden globally. However, how the global distribution of water-associated infectious pathogens/diseases looks like and how such distribution is related to possible social and environmental factors remain largely unknown. In this study, we compiled a database on distribution, biology, and epidemiology of water-associated infectious diseases and collected data on population density, annual accumulated temperature, surface water areas, average annual precipitation, and per capita GDP at the global scale. From the database we extracted reported outbreak events from 1991 to 2008 and developed models to explore the association between the distribution of these outbreaks and social and environmental factors. A total of1,428 outbreaks had been reported and this number only reflected ‘the tip of the iceberg’ of the much bigger problem. We found that the outbreaks of water-associated infectious diseases are significantly correlated with social and environmental factors and that all regions are affected disproportionately by different categories of diseases. Relative risk maps are generated to show ‘hotspots’ of risks for different diseases. Despite certain limitations, the findings may be instrumental for future studies and prioritizing health resources.

## Introduction

Although substantial advances in biomedical sciences and public health measures have facilitated control of many infectious diseases in the past century, the world has witnessed an increasing incidence and geographical expansion of emerging and re-emerging infectious diseases [1], which, together with some other old ones, remain among the leading causes of deaths and disability worldwide [2], [3]. The global environmental, ecological, and socio-economic changes have a significant impact on the distribution, emergence and re-emergence of infectious diseases and are expected to continue to influence such trend [1], [4], [5], [6], [7], [8], [9]. Some recent studies at both global and regional scales have suggested that climatic factors, human movement, and agricultural practices are important factors underlying the distribution, emergence, and re-emergence of infectious diseases [1], [6], [10].

Water is essential for maintaining life on Earth. Meanwhile, water can also serve as a media for hazardous substances and pathogenic organisms, posing substantial health threats to humans through a variety of pathways. During the past few decades, human development, population growth, extreme weather events, natural calamities, and climate change have exerted many diverse pressures on both the quality and quantity of water resources which may in turn impact conditions fostering water-associated diseases. Worldwide, water-associated infectious diseases are a major cause of morbidity and mortality [11], [12], [13]. A conservative estimate indicated that 4.0% of global deaths and 5.7% of the global disease burden (in DALYs) were attributable to a small subset of water, sanitation, and hygiene (WSH) related infectious diseases including diarrheal diseases, schistosomiasis, trachoma, ascariasis, trichuriasis, and hookworm infections [11], [14], [15]. Although unknown, the actual disease burden attributable to water-associated pathogens is expected to be much higher. A total of 1415 species of microorganisms have been reported to be pathogenic, among which approximately 348 are water-associated, causing 115 infectious diseases [5].Yet, their distribution and associated factors at the global scale remain largely unexplored.

Although the linkage between the hydrological cycle and infectious diseases has long been recognized, the underlying mechanisms shaping this relationship at global and regional scales are rarely characterized. Recent developments in hydrology and geo-spatial technology, and increasing availability of spatial socio-environmental information provide an opportunity to explore this issue. Geospatial techniques (e.g. Geographic Information System, or GIS, and spatial analytical techniques) offer a means for developing and organizing spatially explicit information. For example, the availability of information on terrestrial surface water area from the Global Lakes and Wetland Database [16], could allow the exploration of the possible relationship between the availability of terrestrial surface water and distribution of water-associated diseases at the global scale.

In this study, a comprehensive database has been developed for global water-associated infectious pathogens and diseases and socio-environmental information which have been integrated into a GIS database. The overall goal of our study is to explore the possible relationship between global distribution of water-associated infectious diseases and socio-environmental factors. In this study reported outbreaks of water-associated diseases were chosen as the study subject as they were available in the developed database and provided semiquantitative information (e.g. yes or no, and frequency of outbreaks). Our specific aims in this study were to describe the global distribution of reported outbreaks caused by water-associated infectious diseases from 1991 to 2008, to explore potential risk factors associated with spatio-temporal distributions of these outbreaks, and to develop a global risk map for these diseases.

## Methods

### 1. Disease database development

Primary source of information on water-associated pathogens and infectious diseases for the database developed in present study was based on the Global Infectious Disease and Epidemiology Network (GIDEON), a subscription- and web-based comprehensive global infectious diseases database which provides extensive geographical and epidemiological information including outbreaks for 337 recognized infectious diseases in 231 countries and regions. Data in GIDEON are collated through a system of computer macros and dedicated source lists developed over the past 15 years. A monthly search of Medline is conducted against a list of GIDEON key words (similar to Mesh terms in PubMed), and titles/abstracts of interest are reviewed. In addition, all standard publications of WHO and CDC are scanned for relevance before they are collated and entered into GIDEON. The GIDEON infectious diseases database provides a chronological listing of all reported outbreaks of infectious diseases, which are listed by year and country, with specific location information available for the majority of reported outbreaks. For those without specific location information, original publications or reports were searched to extract the information. To assess GIDEON's completeness on the reported outbreaks, a systematic search based on PubMed, ISI Web of Knowledge, WHO and CDC reports was conducted on reported outbreaks (1991–2008) for 10 randomly chosen water-associated diseases. Search terms included names of specific pathogen(s)/disease(s) and country/region, “outbreak”, “epidemic”, and “epidemics”, respectively. Chi-square test was performed to compare results from the independent search vs. that from GIDEON – our results were largely in agreement with that from GIDEON (*X* ^2^ = 591.2, *P*<0.001). Based on the database developed, water-associated diseases and their corresponding causal agents were systematically reviewed, together with extensive literature review for relevant environmental, biological, and epidemiologic characteristics. For each disease, the following information was included in the database we developed.

1. Taxonomic group of causative agents. Five general groups were included - bacteria (including rickettsia), virus (including prions), fungi, protozoa, and helminthes (including cestodes, nematodes, trematodes and acanthocephalans).
2. Water mediation of the disease transmission. Following a general framework on the classification of water-associated infectious disease [17], each disease was classified into one of the following five categories: water-borne, water-based, water-related, water-washed, and water-dispersed. Water-borne diseases, such as typhoid and cholera, are typically caused by enteric microorganisms, which enter water sources through fecal contamination and cause infections in humans through ingestion of contaminated water. To account for water-borne pathogens (e.g. *Cryptosporidium, Giardia*) whose transmission can be through accidental ingestion of, or exposure to, contaminated water in recreational settings (for example), we identified outbreaks caused by this transmission pathway and included them in “water-carried diseases”, a sub-group of water-borne diseases by following Steiner et al. [21]; water-based diseases commonly refer to diseases caused by infections of worms which must spend parts of their life cycles in the aquatic environment, such as schistosomiasis; water-related diseases, such as malaria and trypanosomiasis, need water for breeding of insect vectors to fulfill the transmission cycle; water-washed diseases are those whose transmission is due to poor personal and/or domestic hygiene as a result of lack of appropriate water; and finally, water-dispersed diseases are caused by infections of agents which proliferate in fresh water and enter the human body through the respiratory tract, such as *Legionella*.
3. Transmission routes. Based on the process and nature of transmission, each disease was assigned to one of the four primary transmission groups following the framework by Eisenberg et al. [4]: directly transmitted, vector-borne , environmentally-mediated, and zoonotic. The directly transmitted diseases are those primarily caused by pathogens transmitted via person-to-person contact, where “contact” between humans is the principle mode of transmission, either through intimate proximity (e.g. droplet spray) or bodily fluid exchange. In this group, humans are the only host and the environment typically does not serve as reservoir for the pathogens. Vector-borne diseases are caused by pathogens which are carried by vectors (e.g. mosquitoes) and transmitted to humans through biting. For environmentally-mediated diseases, the environment (e.g., food, water and soil) plays a significant role in a pathogen's life cycle and transmission occurs between humans and the environment directly or indirectly. The zoonotic transmission diseases are diseases that are naturally transmitted between vertebrates and humans. For diseases which may have more than one transmission route, their primary transmission route was used in the database.
4. Outbreak events and emergence/re-emergence of water-associated infectious diseases. In the database an outbreak was defined as an increase in cases of disease above what was normally expected in that population in that area and a reported outbreak referred to an outbreak that was reported. Reported outbreaks of water-associated diseases between 1991 and 2008 were extracted from the database. For each outbreak, information including the causal agent, time, and location of the outbreak was extracted from the database. Most of the reported outbreaks had location information (e.g. villages, counties, or cities where the outbreaks took place). For those without location documented in the database (GIDEON), original publications or reports were checked to retrieve outbreak locations. For reported outbreaks, the spatial scales of reports obtained were on the order of municipality/county or smaller. Based on centroid points of geographical areas (e.g., village, county, or city) where outbreaks were reported, the outbreaks were positioned in Google Earth® and corresponding longitudinal and latitudinal information were extrapolated to ArcGIS (9.2) for grid-based (one degree) analyses described below. For causal agents of the outbreaks, they were also characterized as either emerging/re-emerging or non-emerging pathogen(s) by following the criteria previously defined [5].

### 2. Socio-environmental database

The database included the following information - grid-based global human population density (per km^2^) based on the 2000 global population dataset, which was developed by Socioeconomic Data and Applications Center (SEDAC) of Columbia University between 2003 and 2005, providing globally consistent and spatially explicit human population information (http://sedac.ciesin.columbia.edu/gpw/); global average accumulated temperature (degree days, with a spatial resolution of 0.5 degree) for the period between 1961 and1990 from United Nation Environmental Protection(http://www.unep.org/), which was based on the degree that the temperature rose above zero degree and the number of days in the period during which this excess was maintained [18]; surface area (km^2^) of water bodies including large lakes , rivers, and wetland, collected from the global lakes and wetlands database (http://www.worldwildlife.org/); the average rainfall (mm) per year for the period between 1961 and1990 from FAO (http://www.fao.org); and per capita Gross Domestic Product (GDP) which was based on each a country's GDP divided by the total number of people in the country ( http://sedac.ciesin.columbia.edu/ddc/baseline/). The scale of all information collected was converted to one-degree grid in the GIS database.

### 3. Statistical analyses

#### Controlling for reporting bias

**Figure 1:**
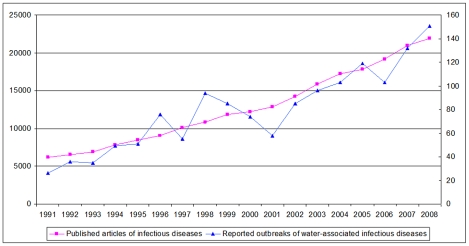
Trends in published articles on infectious diseases and reported outbreak events of water-associated infectious diseases. Shown are the global trends in the number of publications on infectious disease and reported outbreaks of water-associated infectious diseases from 1991 to 2008 (Pearson correlation - 0.935, *P<0.001*).

Primary source of information on reported outbreak events was from GIDEON, which is based on peer-reviewed publications and reports of governmental and international agencies (e.g. CDC and WHO), and considered comprehensive. However, as long being recognized, underreporting of infectious disease outbreaks widely exists, depending on a number of factors such as a country's socio-economic status and investment of research resources. For instance, outbreak events are more likely to be reported in developed countries than in developing countries due to greater availability of resources in the former, which may cause reporting bias [19]. To account for the potential bias, reporting efforts for each country were quantified by estimating published articles specifically related to each country from 1991 to 2008 following Jones et al.'s approach [1]. Using PubMed, “infectious disease” and “country name” were used as keywords in the search of publications to approximate the reporting efforts for each country. Figure 1 shows the global trend in the number of publications on infectious diseases and reported outbreaks of water-associated infectious diseases from 1991 to 2008, suggesting a strong correlation between the outbreak events and publications. In the analysis, the number of publications for each country (e.g. grid cells within each country having the same number) was set as an offset variable to control for reporting bias [1].

#### Descriptive/exploratory analysis

Basic characteristics (e.g. taxonomy and transmission routes) of causal agents associated with the outbreak events were summarized. Temporal trend of outbreak events in relation to the number of publications was tested. Exploratory analysis was conducted using a multivariable logistic regression to control for co-variability between independent variables, with the presence/absence of outbreak events as the dependent variable and all factors including the reporting effort by each country as independent variables. Correlation analyses were conducted for socio-economic variables including population density, global accumulated temperature, per capita GDP. The variables of statistical significance in the correlation analyses were included in the Bayesian analysis described below. All analyses were performed using SPSS (SPSS Inc., USA).

#### Bayesian analysis

The global terrestrial area was divided to 17,597 1×1 degree grid cells using ArcGIS software (ESRI, USA). The grid-based socio-environmental data were matched to the grid cells using the spatial module of ArcGIS. For grid cells overlapping international borders, the grids were allocated to the countries where the outbreaks were reported (no outbreaks from multiple countries falling in one grid were found in the database). The reported outbreak events were then counted for each grid cell for the period from 1991 to 2008. Due to excessive zero counts in many cells, zero-inflated Poisson regression models – one without and another with spatial structure - were developed to explore risk factors that may be associated with the outbreaks. In the first model with no spatial structure, the count of outbreak events in one grid cell, 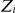 in country *j*, was assumed to follow a zero-inflated Poisson distribution (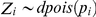) [20], and socio-environmental variables as 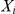, the 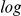 of 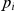 can be expressed as, 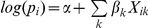. The second model integrated spatially structured random effects of the countries and the model was given as 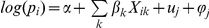, where 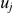 and 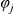 were random effects representing spatially unstructured and structured heterogeneity between countries. The spatial effect 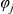 was assumed from a conditional autoregressive model (CAR), which implied that each 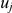 was conditional on the neighboring 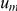, and followed a normal distribution with the mean equal to the average of the neighboring 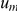. The model parameters were estimated using Bayesian inference by employing Markov chain Monte Carlo simulation. A single chain sampler with a burn-in of 15, 000 iterations was run. The inference of the parameters was based on 10,000 iterations after the burn-in phase. The deviance information criterion (DIC) was used to assess the goodness of fit of models [21], [22] and the model with the smallest DIC was considered to be the best fit. All regression coefficients and associated 95% Bayesian credible intervals (95% BCI) were computed via the Gibbs sampler. The Bayesian analysis was conducted in WinBUGS 1.1.4 (MRC Biostatistics Unit, Cambridge, UK).

#### Risk predictions and mapping

The relative risk for each category of water-associated diseases at each grid cell was estimated by the exponent of 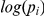 using the best fit models, e.g. if the spatial model was the best model, the relative risk was estimated by exponent of 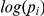, 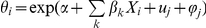, otherwise 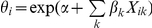. Standard deviation (SD) was used to approximate analysis uncertainty. Based on the estimates of each category of water-associated diseases for each grid, maps were generated to show global distributions of relative risks of water-associated outbreaks and prediction uncertainties.

## Results

**Figure 2:**
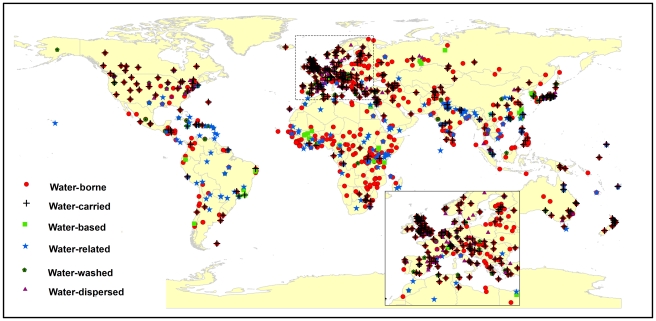
Distribution of reported outbreaks of water-associated infectious diseases from 1991 to 2008.

A total of 1,428 outbreak events had been reported from 1991 to 2008. Outbreaks occurred all over the world and the clusters of reported outbreaks tended to be in west Europe, central Africa, north India and Southeast Asia (Figure 2). Among the reported outbreak events, 70.9% (1,012) were associated with water-borne diseases including 32.9% (471) water-carried, 12.2% (174) water-related, 6.8% (97) water-washed, 2.9% (41) water-based, and 7.3% (104) water-dispersed. 46.7% (667) of the outbreak events were associated with emerging or reemerging pathogens, which appeared in humans for the first time or had occurred previously but were increasing in incidence or expanding into areas where they had not previously been reported [5]. It is found that 49.6% (709) of the outbreak events was caused by bacteria, 39.3% (561) by viruses, and 11.1% (158) by parasites. 6.5% (93) of the outbreak events was caused by agents that could be transmitted by direct contact, 1.1% (16) transmitted through vectors, 63.5% (907) through environmental transmission, and 28.9% (412) by zoonotic routes.

The reported outbreak events had shown a significant increase since 1991, which had been accompanied by a significant increase in the number of published articles (Figure 1, Pearson correlation - 0.935, *P<0.001*). We used a generalized linear model to test the temporal trend in the outbreak events and found it insignificant (t = 0.046, *P = 0.940*) after controlling for the publication efforts. The number of published articles was therefore used as a covariate in the subsequent statistical analyses.

Table 1 summarizes analyses of the Poisson models without and with spatially structured random effects using Bayesian inference for the five categories of water-associated diseases. The DIC values of the Poisson model with spatial random effects are smaller than that without spatial structure, suggesting that the spatial models provided a better fit to the data. The Poisson models with spatial structure were therefore used for risk factor analysis and mapping.

**Table 1:** Parameter estimates of the Poisson models with Bayesian inference (posterior mean with 95% credible intervals).

Variables	Water borne diseases		Water-carried disease		Water-washed disease	
	model 1^a^	model 2^b^	model 1	model 2	model 1	model 2
Constant	−0.345(−1.587, 1.439)	−0.367(−0.824,0.738)	−4.120(−4.303,−3.849)	−2.389(−3.211,−1.539)	−4.34(−6.898,−3.327)	−2.373(−2.373,−0.454)
Population density	0.231(0.134,0.691)	0.140(0.119,0.162)	0.215(0.185,0.239)	0.177(0.142,0.210)	0.213(0.129,0.268)	0.239(0.239,0.344)
Accumulated temperature	0.137(0.041,0.321)	0.141(−0.035,0.330)	−0.079(−0.206,0.039)	−0.079(−0.322,0.158)	−0.067(−0.569,0.431)	−0.511(−0.511,−0.066)
Water area	−0.112(−0.391,0.031)	0.047(−0.069,0.047)	−0.142(−0.318,−0.003)	−0.052(−0,121,0.183)	−4.568(−6.981,−1.671)	−4.197(−4.197,−1.849)
Average rainfall	−0.133(−0.125,−0.056)	−0.322(−0.618,−0.132)	−0.193(−0.543,−0.030)	−0.431(−1.047,−0.431)	−0.071(−0.431,0.114)	−0.567(−0.567,−0.13)
DIC^c^	7643.12	5893.89	2749.240	2121.870	1125.519	876.671

Variables	Water-based diseases		Water-related diseases		Water-dispersed diseases	
	model 1	model 2	model 1	model 2	model 1	model 2
Constant	−6.135(−7.476,−5.396)	−5.699(−8.199,−2.651)	−0.145(−3.966,,−1.523)	−1.072(−2.295,−0.512)	−5.891(−6.113,−4.171)	−6.693(−9.033,−5.309)
Population density	0.212(0.124,0.389)	0.261(0.137,0.386)	0.128(0.049,0.232)	0.145(0.122,0.168)	0.256(0.215,0.286)	0.301(0.207,0.407)
Accumulated temperature	0.431(0.281,0.951)	0.551(−0.183,1.403)	0.149(0.131,0.671)	0.157(−0.016,0.333)	−0.345(−0.583,−0.133)	0.113(−0.485,0.634)
Water area	−0.006(−0.765,0.017)	0.013(−0.773,0.512)	−0.009(−0.626,0.091)	0.04(−0.084,0.163)	−0.304(−0.74,−0.008)	−0.009(−0.42,0.235)
Average rainfall	−0.567(−2.791,0.678)	−0.703(−3.497,0.179)	−0.309(−1.831,0.321)	−0.317(−0.583,−0.137)	−0.068(−0.408,0.125)	−0.146(−0.514,0.095)
DIC	560.709	459.017	6456.567	5987.160	1241.680	976.165

a Model 1 is the simple Poisson model without spatially structured random effects.

b Model 2 is the Poisson model with spatial random effects.

c DIC is deviance information criterion and the smaller value of DIC indicates a better fitting model.

The population density was shown to be a significant risk factor for reported outbreaks of all categories of water-associated infectious diseases and the probability of outbreak occurrence increased with the population density. The accumulated temperature was a significant risk factor for water-related diseases only. The analysis suggested that occurrence of water-washed diseases had significantly inverse relationship with surface water areas. Such inverse relationship was also observed between the average annual rainfall and water-borne diseases (including water-carried) and water-related diseases.

**Figure 3:**
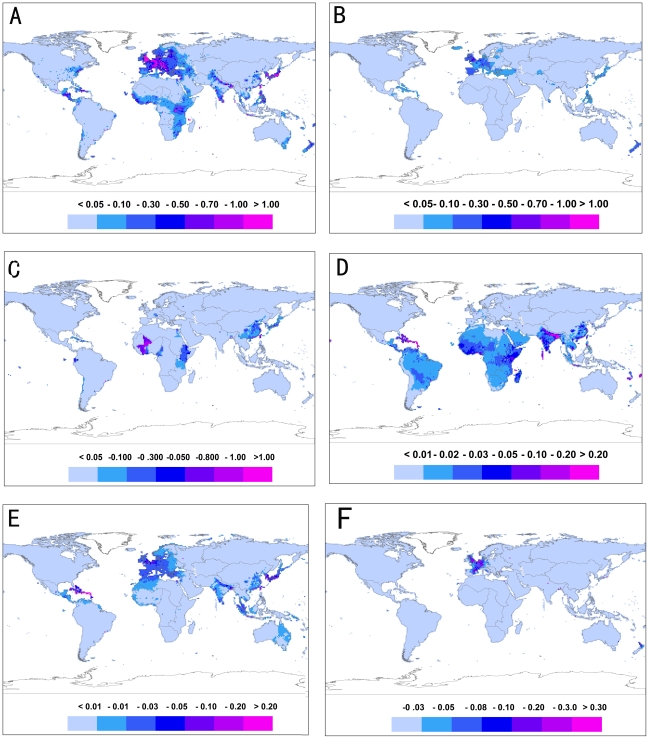
Relative risk of water-associated infectious diseases. Shown are relative risk distributions for different categories of water-associated infectious diseases – water-borne (A), water-carried (B), water-based (C), water-related (D), water-washed (E), and water-dispersed (F). Relative risk estimate was based on the best fit Bayesian model integrating reported outbreaks, random and spatial effects.

Figure 3 (A–F) shows the risk distribution based on the model predictions with the blue indicating lower risk while the red representing higher risk. The model predictions suggested that west Europe, central Africa, north India were at the higher risk for water-borne diseases (e.g. *Escherichia coli* diarrhea), and notably, that the higher risk for water-borne diseases in west Europe was primarily driven by water-carried diseases (e.g. cryptosporidiosis). West Europe, North Africa, and Latin America tended to be at higher risk to water-washed diseases (e.g. viral conjunctivitis). Risks associated with water-based diseases (e.g. schistosomiasis) were higher in east Brazil, northwest Africa, central Africa, and southeast of China. High risk areas for water-related diseases (e.g. malaria and dengue fever) were clustered in central Africa in particular Ethiopia and Kenya, and north India. For water-dispersed diseases (e.g. Legionellosis), west Europe seemed to be at higher risk.

## Discussion

In the past decade there has been an increasing interest in understanding factors underlying the distribution of infectious pathogens, emerging and re-emerging infectious diseases. Some recent research efforts have been in attempt to determine large-scale ecological factors associated with diversity richness and distribution of infectious and parasitic pathogens [6], socio-environmental determinants of emerging infectious disease [1], and to explore the impact of global environmental change on distribution and spread of infectious diseases [23], [24]. These studies have offered valuable insights into understanding socio-environmental processes and factors underlying the distribution of infectious diseases. In this study, we focused our attention on water-associated infectious diseases and attempted to explore whether these diseases follow similar patterns observed in other studies [1], [6], and whether the distribution and occurrence of these diseases were related to terrestrial water dynamics (e.g. precipitation and land-surface water) together with other socio-environmental factors. The transmission of many infectious diseases is closely linked to water and the water-infectious pathogen interactions exhibit a complicated relationship depending on the transmission characteristics of the pathogens and water's roles in the transmission. The study showed that water-associated infectious diseases and outbreaks were broadly distributed throughout the world but the distribution of specific agents/diseases varied greatly from region to region. The majority of reported outbreaks events were associated with water-borne pathogen including those water-carried. Water-borne diseases have a much broader distribution than other water-associated diseases, suggesting a broader impact of waterborne pathogens in particular those related to fecal-oral route and water, sanitation, and hygiene. In addition to water, other environmental factors have also been recognized to play a significant role in the distribution, transmission, and outbreaks of these water-associated diseases [25], [26], [27].

It should be noted that, though, the outbreaks reported here only reflected “the tip of the iceberg” of the much larger problem. A complete count of outbreaks attributable to water-associated pathogens is impossible as underreporting is a universal problem, and reporting efforts and effectiveness may vary from country to country, and pathogens to pathogens, depending on many factors particularly availability of research and surveillance resources, and epidemiological characteristics of causal agents. In developing countries, outbreaks of many vector-borne infectious diseases such as dengue and malaria [28], [29] and gastrointestinal infections [30] were grossly underreported, partly due to their endemic characteristics. Even in the US, reporting completeness of notifiable infectious diseases varied from 9% to 99%, and was strongly associated with diseases being reported [31]. In general, water-borne pathogens usually exhibit acute manifestations and are more likely to be reported [32]. In contrast, other diseases such as water-based schistosomiasis, a disease of chronic infections and atypical symptoms, are more likely to be underreported. In this study, the primary source of outbreak information was from GIDEON, which is the most comprehensive database on infectious diseases and offers detailed information on epidemiology including distributions and outbreaks of infectious diseases for more than 205 countries and regions, as well as clinical manifestations and treatment associated with each disease [23], [33]. As expected, GIDEON does not include all outbreak information due to underreporting of outbreak events, but we believe that information from GIDEON is representative and provides an overview of available and recognized outbreak data, as argued by some other studies [23], [33].

The distribution of water-associated diseases, like many other infectious diseases, is highly heterogeneous. The spatial structure associated with the distribution of the outbreaks may be important in understanding underlying risk factors. To explore possible associations between socio-environmental factors and the outbreaks at the global scale, two Poisson models (without and with spatial structures) were developed. Among the two models explored, the one incorporating spatial effects provided a better fit to the data. Our findings suggested that the importance of these socio-environmental variables was dependent on the category of water-associated diseases. Human population density was a common significant risk factor for the outbreaks caused by all categories of water-associated diseases, in concurrence with the previous study suggesting that human population was an important predictor of emerging infectious diseases event at the global scale [1]. The accumulated temperature was a significant factor associated with water-related diseases, which was in agreement with many other studies [34], [35], [36], [37]. The transmission of diseases in this category typically involves vectors (e.g. mosquitoes) which require certain energy level (e.g. accumulated temperature) allowing completion of development of vectors and pathogens [10], [38], [39]. In this study, terrestrial surface water area (at each grid-region) was found to be inversely proportional to the outbreak events associated with water-washed diseases such as trachoma. The primary determinant of water-washed diseases is poor personal and/or domestic hygiene typically due to insufficient sanitary water for hygienic purpose, and this has been reported in many site-specific studies [40], [41], [42]. Our result from a large-scale correlation study supported these points of the previous studies, suggesting that regional water availability may be indicative of local water availability which is closely linked to personal and domestic hygiene. Our analysis indicated a negative relationship between average annual rainfall and water-related diseases, in contrast with some previous studies showing that some outbreaks of water-related diseases are positively associated with heavy rainfall events [8], [43], [44], [45]. This can be partly explained by issues related to scale and timing effects – the majority of studies reporting positive relationship between precipitation and waterborne illness was conducted at local scale and typically time-lag effects were considered. Indeed, the rainfall and water-related diseases exhibit complex relationships as shown in previous studies, and many rainfall-driven transmission and outbreaks were dependent on local circumstances. In addition to rainfall, multiple and covarying drivers have also been proposed for seasonal pattern of transmission and outbreaks of many water-associated diseases, including temperature, host demographic and biological characteristics [46], [47], [48]. However, due to lack of global information on seasonal patterns of outbreaks and the driving factors, temporal heterogeneity of outbreaks events, such as seasonality discussed here, was not included in the present study.

Using the best-fitted models we predicted global distributions of relative risks associated with each category of water-related infectious diseases, as shown in Figure 3. Surprisingly, the risk maps show that west Europe and central Africa were all at relatively higher risk for water-borne diseases. A closer look at pathogens associated with the reported outbreaks indicated different dominant species in the two regions – in Africa reports of water-borne outbreaks were primarily associated with *Vibrio cholerae*, whereas in west Europe *giardia, cryptosporidium* were common in the water-borne outbreaks, with the latter being particularly related to accidental ingestions of contaminated water (e.g. in recreational settings) and, to some extent, mixed with infections of food-borne sources [49], [50], [51].

Some limitations of the current study are recognized. Although possible reporting bias was adjusted for using publications for each country, the analysis may have missed countries/regions with outbreaks but no publications and/or reports. Second, only a few socio-environmental factors were considered in the present study and it is likely that some other factors might be associated with the outbreaks. In addition, significant prediction uncertainties were noted throughout the outbreak countries and regions, this was partly due to the temporal correlation of the outbreak events which was not considered in the analysis. The addition of such information (e.g. temporal trend of outbreaks in places where repeated outbreaks occurred) to the model may improve model prediction. In spite of these, we think that overall patterns of distribution and associated risk factors presented here are informative and offer insights into global distribution and risk factors associated with water-associated diseases, although further studies on other possible risk factors and modeling approaches to improving prediction are still needed.

In conclusion, our study, to our knowledge, is the first to describe global distribution of outbreaks caused by water-associated infectious diseases and explore possible risk factors underlying the distribution of these outbreaks at the global scale. The risk maps may offer insights for future studies and for prioritizing health resources.
